# Study on the Control Method of Knee Joint Human–Exoskeleton Interactive System

**DOI:** 10.3390/s22031040

**Published:** 2022-01-28

**Authors:** Zhipeng Wang, Chifu Yang, Zhen Ding, Tao Yang, Hao Guo, Feng Jiang, Bowen Tian

**Affiliations:** 1 School of Mechatronics Engineering, Harbin Institute of Technology, Harbin 150001, China; 19B908078@stu.hit.edu.cn (Z.W.); cfyang@hit.edu.cn (C.Y.); 16b908060@stu.hit.edu.cn (Z.D.); 20b908071@stu.hit.edu.cn (T.Y.); 2 School of Computer Science and Technology, Harbin Institute of Technology, Harbin 150001, China; guohao@hit.edu.cn; 3 School of Business Administration, Zhongnan University of Economics and Law, Wuhan 430000, China; callytian1128@alumni.hust.edu.cn

**Keywords:** exoskeleton, force loading, disturbance observer, human–exoskeleton interaction, angle prediction

## Abstract

The advantages of exoskeletons based on the Bowden cable include being lightweight and flexible, thus being convenient in assisting humans. However, the performance of an exoskeleton is limited by the structure and human–exoskeleton interaction, which is analyzed from the established mathematical model of the human–exoskeleton system. In order to improve the auxiliary accuracy, corresponding control methods are proposed. The disturbance observer is designed to compensate for disturbances and parameter perturbations in the inner loop. The human–exoskeleton interaction feedforward model is integrated into the admittance control, which overcomes the limitation of the force loading caused by the friction of the Bowden cable and the change in stiffness of the human–exoskeleton interaction. Furthermore, an angle prediction method using the encoder as the signal source is designed to reduce the disturbance of the force loading caused by human motion. Finally, the effectiveness of the design method proposed in this paper is verified through experiments.

## 1. Introduction

Exoskeletons have been extensively studied in recent years because they can enhance the athletic ability of humans. According to different structures, the lower-extremity exoskeletons can be divided into rigid exoskeletons [1,2,3] and flexible exoskeletons [4,5,6]. The mechanism of a rigid exoskeleton is parallel to the human limbs, which is convenient for weight bearing and rehabilitation training. The corresponding control methods [7,8] have also been studied. However, due to structural and inertial limitations, rigid exoskeletons are unsatisfactory in assisting healthy individuals to walk. Therefore, a wearable, flexible exoskeleton is proposed. The flexible exoskeleton mainly comprises textiles and the Bowden cable [4]. The textile is the bearing point of the sheath of the Bowden cable. The Bowden cable [9,10] has advantages such as long transmission distance and flexible layout. Therefore, the actuator can be concentrated near the center of body weight. The integration and flexibility of the exoskeleton can be effectively enhanced.

The human–exoskeleton system (HESY) contains two inputs and one output [11]: a force command, active human motion, and force response. In [12], the HESY’s parameter perturbation and active movement are regarded as disturbance. In other words, the HESY is regarded as a single-input and single-output system. A disturbance observer (DOB) is designed to compensate for the disturbance caused by parameter perturbation and active motion based on the dynamic model. To further improve the dynamic performance of the force loop, a sliding mode controller based on the nominal model [12,13] is proposed. However, human movement is regarded as a disturbance [12], which is not conducive to the coordination of the HESY.

Some research has been carried out based on the combination of motion compensation and controller design. Some studies predefined the joint motion trajectory [14]. However, the gait of the human is not entirely consistent. A new compensation method has been proposed [15] to avoid fixed motion trajectories, which slowly swings the hip joint under a preload of 15 N while recording the position relationship between the IMU angle and the motor encoder. The mapping relationship is used to compensate for the active movement of the human. However, this method requires calibration before each wear. Moreover, the force is generated before the change in displacement. This method cannot predict the changes in human joints in advance. Therefore, the active movement of the human will interfere with the assistance. This phenomenon has also been verified in this paper. Therefore, it is necessary to study the method of motion prediction. The video posture coding method [16] has been proposed to predict the angle trajectory of human joints in the sagittal plane. This method relies on three cameras to collect signals, and the shooting angle has a more significant impact on the prediction accuracy. Thus, the application scenarios of this method are limited. The surface electromyography signal (sEMG) is related to the degree of muscle activation. Moreover, the change in sEMG can be 40–100 ms earlier than the change in human motion [17]. The sEMG and joint angle regression model is established through deep belief networks and back propagation (BP) neural networks [18]. To further improve the prediction effect, sEMG, joint angles, and plantar pressure signals [19,20] are introduced into the generalized regression neural network for training and prediction. Similarly, sEMG and A-mode ultrasound [21] have been combined and introduced to build a vector machine regression model. Although the above experimental results prove the effectiveness of the prediction method, the effectiveness of predicting the joint angle in advance to reduce the disturbance has yet to be verified. In addition, the neural network has a large number of calculations and it is difficult to ensure the real-time performance required by the lower-level control.

The inverse dynamic model has been established [22,23] and the torque required to assist the human is calculated based on the motion information. The servo motor adopts the torque mode and the calculation result is directly used as the torque command of the servo motor. The accuracy of this method depends on the estimation of the model and other interference. Studies have shown that more than 50% of the torque output by the servo motor is used to overcome the friction of the Bowden cable [24], which is accompanied by hysteresis. Therefore, the inaccurate estimation of friction affects the assisting effect. Zhang [14] adopted a force loop control frame based on the position of the inner loop to overcome friction and fixed trajectory interference. Proportional control and damping injection are used in the force loop. The force error that still exists in one gait cycle is compensated for by iterative control in the next gait [25,26]. To ensure the compliance of human–exoskeleton interaction (HEI), the admittance control framework based on the inner loop was established [27,28]. The stiffness of the HEI changes significantly during the assisting process. Therefore, closed-loop control can only compromise on rapidity to attain the stability under the condition of minimum stiffness. The changing relationship between force and displacement was fully utilized to reduce the influence of excessive stiffness changes on force loading [29,30]. On the one hand, it can avoid the limitation of low stiffness to the entire system’s performance. On the other hand, applying a force–displacement relationship instead of a force closed-loop can effectively reduce the disturbance caused by the hysteresis phenomenon of the Bowden cable.

The main contribution of this paper is to propose corresponding control strategies to reduce the influence of the exoskeleton structure and HEI on human assistance. This paper adopts the admittance control framework based on the speed inner loop to realize the assistance in the active movement. The DOB is designed in the speed inner loop, which can enhance the dynamic performance of the inner loop under time-varying conditions. The human–exoskeleton interaction feedforward model (HEIF) is added to the admittance control to compensate for Bowden cable friction disturbance and the HEI’s stiffness change on the force loading. Finally, an angle prediction method that adopts the encoder as the signal source is proposed, effectively reducing the influence of human motion and signal acquisition delay on force loading.

The structure of this paper is as follows. The mathematical model of the HESY is established in Section 2. The corresponding controller design is presented in Section 3. The experimental verification of the theory in this paper is shown in Section 4. The conclusions are presented in Section 5.

## 2. Mathematical Modeling of the HESY

**Figure 1:**
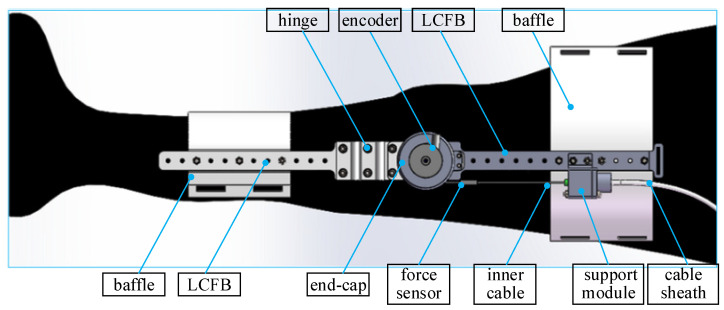
The fixation mechanism of the knee joint [31].

The farther the distance between the load and the center of body weight, the more metabolism the body consumes. Therefore, the power supply, controller, and servo motor are integrated near the center of body weight. The fixation mechanism of the knee joint [31] is shown in Figure 1, which is driven by a servo motor through a Bowden cable. Two lightweight carbon fiber boards (LCFB) and hinge mechanisms can realize rotation in the sagittal and coronal planes. The servo motor is set to speed mode to improve the dynamic response of the inner loop.

**Figure 2:**
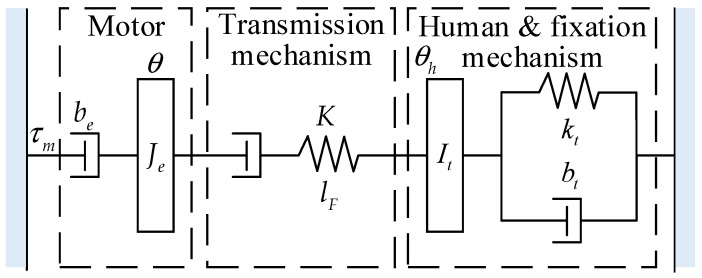
The fixation mechanism of the knee joint.

The schematic diagram of the HESY is shown in Figure 2. According to Figure 2, the mathematical model of the HESY can be established.

$\tau_{m}$ is the output torque of the current loop of the servo motor. $J_{e}$ and $b_{e}$ are the equivalent moment of inertia and equivalent damping of the servo motor, respectively. θ is the angular displacement of the input shaft of the servo motor reducer. K and $l_{F}$ are the comprehensive stiffness and elastic deformation of the transmission mechanism between the servo motor and the knee joint fixation mechanism, respectively. $\theta_{h}$ is the angle of the knee joint encoder. *I_t_* = *I_h_* + *I_l_*, where $I_{h}$ and $I_{l}$ are the equivalent inertia moment of the knee joint and fixation mechanism, respectively. $b_{t}=b_{h}+b_{l}$, where $b_{h}$ and $b_{l}$ are the equivalent damping of the knee joint and fixation mechanism, respectively. $k_{t}=k_{h}+k_{l}$, where $k_{h}$ and $k_{l}$ are the equivalent stiffness of the knee joint and fixation mechanism, respectively.

**Figure 3:**
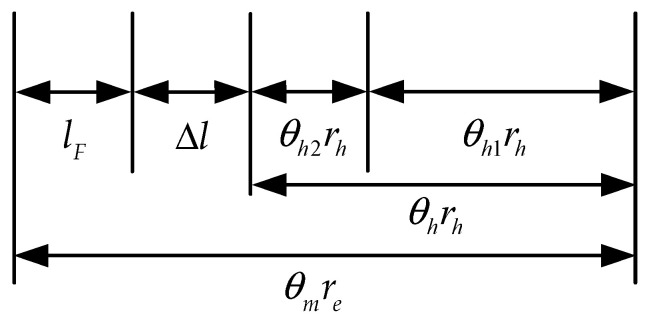
The displacement relationship of the HESY.

Figure 3 shows the displacement relationship of the HESY, as shown below

(1) $$\left\{ \begin{array}{l} \theta_{m}r_{e}=\Delta l+l_{F}+\theta_{h}r_{h}\\ \theta_{h}r_{h}=\theta_{h1}r_{h}+\theta_{h2}r_{h}\\ \theta_{m}=\theta /n \end{array}\right.,$$

where $\theta_{m}$ is the angular displacement of the output shaft of the servo motor reducer, $r_{e}$ is the radius of the sheave at the output shaft of the reducer, Δl is the inelastic deformation during the movement, $\theta_{h1}$ and $\theta_{h2}$ are the angles of the knee joint and the deformation of the HEI, respectively, $r_{h}$ is the radius of the sheave at the knee joint, and n is the reduction ratio of the servo motor.

The mathematical equation of the servo motor speed mode is as follows:

(2) $$G_{2}\left(s\right)=\frac{\dot{\theta }}{{\dot{\theta }}_{d}}=\frac{g}{as^{2}+bs+c},$$

(3) $$\left\{ \begin{array}{l} a=J_{e}L\\ b=J_{e}\left(R+K_{i}\right)+b_{e}L\\ c=b_{e}\left(R+K_{i}\right)+\left(C_{e}+K_{d}K_{v}\right)k_{m}\\ g=k_{m}k_{d}k_{u} \end{array}\right.,$$

where s is the Laplace operator, L is the inductance coefficient, $K_{i}$ is the current feedback coefficient, R is the resistance coefficient, $k_{d}$ is the amplification factor of the speed loop, $k_{u}$ is the amplification factor, $C_{e}$ is the back EMF coefficient, $k_{m}$ is the torque coefficient, and $k_{v}$ is the feedback coefficient of the speed loop. The interaction force is as follows:

(4) $$F=Kl_{F},$$

where F is the interaction force. The knee joint flexion angle in the sagittal plane can reach 60°. Therefore, only the dynamic equation in the sagittal plane is considered. The dynamic equation of the lower limb can be simplified as

(5) $$I_{t}{\ddot{\theta }}_{h}+b_{t}{\dot{\theta }}_{h}+k_{t}\theta_{h}=\tau_{h}+\tau -\tau_{g},$$

where $\tau_{h}$ is the muscle torque of the human, τ is the interaction torque, and $\tau_{g}$ is the gravity torque. Combined with Equations (1)–(5), the block diagram of the inner loop [31] can be obtained as follows.

**Figure 4:**
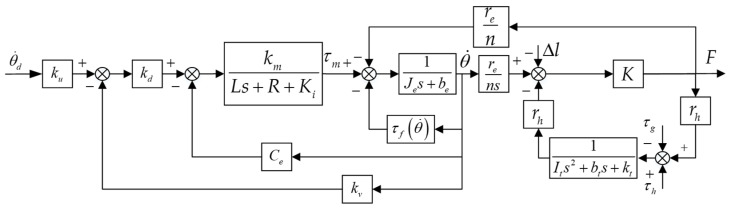
The block diagram of the inner loop [31].

It can be seen from Figure 4 that there are three factors affecting the force loading accuracy. First, the speed loop of the servo motor is disturbed by Bowden cable friction and the interaction force. Due to the poor anti-interference ability of the integrated motor, its dynamic performance is affected. Second, $\tau_{h}$ cannot be accurately measured. Moreover, the impedance characteristic of the knee joint changes with the gait, which enhances the stability of human motion. Therefore, the mathematical modeling of Equation (5) is difficult. Last, the HEI can be deformed during the movement. In this paper, the controller is designed for dealing with the problems mentioned above.

## 3. Controller Design

### 3.1. Design of the Inner Loop Controller

**Figure 5:**
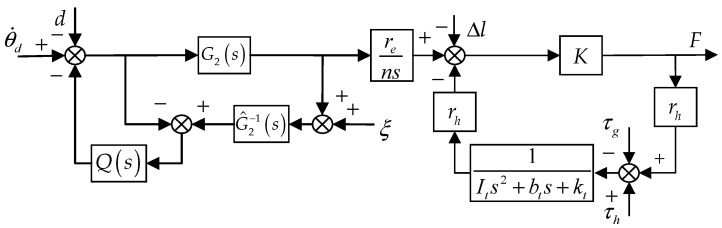
The block diagram of the inner loop based on DOB.

Ensuring the anti-interference ability and stable dynamic performance of the inner loop are the prerequisites for realizing accurate assistance. Mostly, the DOB can observe and compensate for external disturbance and parameter perturbation. A DOB is adopted to improve the dynamic performance of the inner loop. The design of the DOB requires high modeling accuracy. Therefore, the motor speed loop is adopted instead of the knee joint angular velocity closed-loop. The transfer function of the speed loop can be rewritten as

(6) $${\hat{G}}_{2}\left(s\right)=\frac{\hat{g}}{\hat{a}s^{2}+\hat{b}s+\hat{c}},$$

where ${\hat{G}}_{2}\left(s\right)$ is the nominal model of the speed loop. The meanings of the coefficients in Equation (6) are the same as those in Equation (2). The block diagram of the inner loop based on the DOB is shown in Figure 5. Q(s) is the filter and ξ is the high-frequency noise of the speed signal.

The speed loop can be regarded as a three-input one-output system and the transfer function is as follows:

(7) $$\frac{\dot{\theta }}{{\dot{\theta }}_{d}}=\frac{G_{2}\left(s\right){\hat{G}}_{2}\left(s\right)}{{\hat{G}}_{2}\left(s\right)+Q\left(s\right)\left(G_{2}\left(s\right)-{\hat{G}}_{2}\left(s\right)\right)},$$

(8) $$\frac{\dot{\theta }}{d}=\frac{G_{2}\left(s\right){\hat{G}}_{2}\left(s\right)\left(1-Q\left(s\right)\right)}{{\hat{G}}_{2}\left(s\right)+Q\left(s\right)\left(G_{2}\left(s\right)-{\hat{G}}_{2}\left(s\right)\right)},$$

(9) $$\frac{\dot{\theta }}{\xi }=\frac{G_{2}\left(s\right)Q\left(s\right)}{{\hat{G}}_{2}\left(s\right)+Q\left(s\right)\left(G_{2}\left(s\right)-{\hat{G}}_{2}\left(s\right)\right)},$$

(10) $$d=\frac{1}{k_{m}k_{d}k_{u}}\left(\tau_{f}\left(\dot{\theta }\right)+\frac{Fr_{e}}{n}\right).$$

In the low-frequency range, Q(s)=1. On the contrary, Q(s)=0 in the high-frequency range. Therefore, the transfer function of the speed loop can be rewritten as

(11) $$G_{2e}\left(s\right)=\frac{\dot{\theta }}{{\dot{\theta }}_{d}}=\{\begin{array}{ll} {\hat{G}}_{2}\left(s\right) & Q\left(s\right)=1\\ \frac{G_{2}\left(s\right)}{1+Q\left(s\right)\left(G_{2}\left(s\right){\hat{G}}_{2}^{-1}\left(s\right)-1\right)} & 0<Q\left(s\right)<1\\ G_{2}\left(s\right) & Q\left(s\right)=0 \end{array}.$$

It can be seen from Equation (11) that the dynamic performance of the speed loop in the low-frequency band is equivalent to that in the designed nominal model. Both the force disturbance and internal parameter perturbation can be effectively compensated for. However, the bandwidth of the filter is limited by the stability conditions of the DOB. The stability conditions are described as [31]

(12) $${\left\|Q\left(j\omega \right)\right\|}_{\infty }\leq \frac{1}{{\left\|\Delta_{2}\left(j\omega \right)\right\|}_{\infty }}.$$

where $\Delta_{2}\left(j\omega \right)$ is the modeling error of the speed loop. It should be noted that Equation (12) is essential for designing a DOB based on a servo motor instead of knee joints. The inertia and damping of the speed loop can be compensated based on Equation (11). The formula is as follows:

(13) $$G_{2f}\left(s\right)=1+C_{f1}\frac{\hat{a}}{\hat{g}}s^{2}+C_{f2}\frac{\hat{b}}{\hat{g}}s,$$

where $C_{f1}$ and $C_{f2}$ are proportional coefficients, respectively.

### 3.2. Design of the HEIF

**Figure 6:**
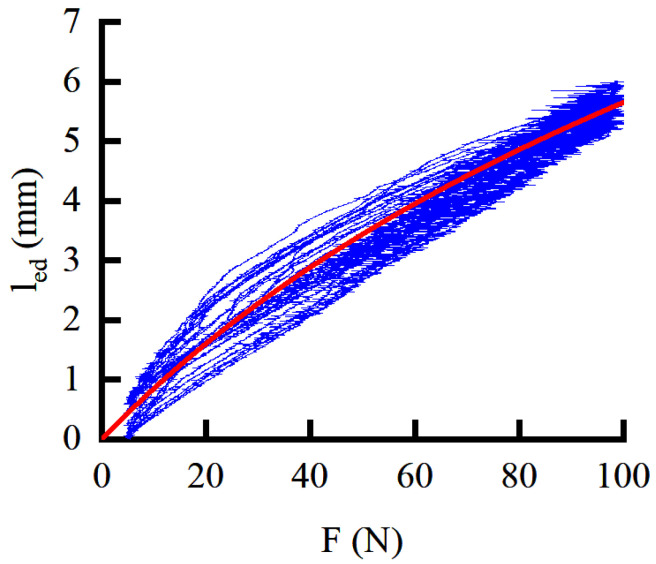
The relationship between the displacement and the interaction force.

Combining Figure 5 and Equation (11), it can be seen that the human motion and the deformation of the HEI can affect the assisting effect. It is necessary to measure the comprehensive stiffness to compensate for the deformation of the HEI. The posture is relatively stable during the initial landing. The motor rotates at a constant speed. During this process, the relationship between the linear displacement of the servo motor and the interaction force is recorded. The measured result is shown in Figure 6. According to the measured data, curve fitting is performed, and the formula is shown follows:

(14) $$l_{ed}=C_{fd1}\ln\left(C_{fd2}F_{d}+1\right),$$

where $C_{fd1}$ and $C_{fd2}$ are the fitting coefficients, respectively. It can be seen from Figure 6 that the HEI has not only nonlinear elastic deformation but also inelastic deformation during the assisting process, which is an inevitable structural feature of the wearable, flexible exoskeleton.

Therefore, to ensure the force loading performance, nonlinear elastic deformation needs to be compensated for as a feedforward term, which is shown below:

(15) $${\dot{\theta }}_{ed}=\frac{nC_{fd1}C_{fd2}}{r_{e}\left(C_{fd2}F_{d}+1\right)}.$$

However, the inelastic deformation cannot be compensated by Equation (15). Moreover, the interaction force needs to be generated by the displacement of the Bowden cable. The inaccurate speed response in the inner loop can cause displacement deviation. Therefore, iterative force control is proposed to compensate for the inelastic deformation of the HEI and the displacement error caused by the speed loop. The iterative speed command of the next gait cycle is corrected by detecting the interaction force error in the current gait cycle. In order to prevent accidental factors from causing excessive force errors and ensure the safety of the system, the maximum single correction displacement is limited to 0.5 mm. The error threshold and speed correction command are as follows

(16) $$\delta_{i}=\left\{ \begin{array}{l} \begin{array}{cc} 0 & \left|e_{F_{i-1}}\right|<e_{F_{0}} \end{array}\\ \begin{array}{cc} 1 & \left|e_{F_{i-1}}\right|\geq e_{F_{0}} \end{array} \end{array}\right.,$$

(17) $${\dot{\theta }}_{ci}=\left\{ \begin{array}{l} \begin{array}{cc} \frac{0.0005}{T_{ei}}n\delta_{i} & \frac{e_{F_{i-1}}}{K}>0.0005 \end{array}\\ \begin{array}{cc} \frac{e_{F_{i-1}}}{KT_{ei}}n\delta_{i} & 0.0005\geq \frac{e_{F_{i-1}}}{K}\geq -0.0005 \end{array}\\ \begin{array}{cc} -\frac{0.0005}{T_{ei}}n\delta_{i} & -0.0005>\frac{e_{F_{i-1}}}{K} \end{array} \end{array}\right.,$$

where $\delta_{i}$ is the speed correction coefficient, $e_{F_{i-1}}$ is the maximum force error value of the previous gait cycle, $e_{F_{0}}$ is the force error threshold value, ${\dot{\theta }}_{ci}$ is the current gait speed correction value, and $T_{ei}$ is the average value of the previous two gait cycles.

### 3.3. Design of the Angle Prediction Method

It can be seen from Equation (1) and Figure 5 that the human motion will interfere with the interaction force. The usual method is to collect the motion information of the knee joint and compensate for it to the servo motor. However, due to factors such as signal delay and phase lag, motion compensation is not timely. The joint angle during walking is a periodic signal. Therefore, parallel oscillators are introduced in this section, and each oscillator learns a particular frequency component of the input signal. In other words, Fourier decomposition is performed on the input signal. Only the fundamental frequency [32] is learned to simplify the design, the others being multiples of it. Each phase of the adaptive oscillator (AO) is as follows:

(18) $${\dot{\phi }}_{i}\left(t\right)=i\omega \left(t\right)+v\theta_{he}\left(t\right),$$

where $\phi_{i}\left(t\right)$ is the phase of the i-th oscillator, ω(t) is the oscillator’s fundamental frequency, $\theta_{he}\left(t\right)=\theta_{h}\left(t\right)-{\hat{\theta }}_{h}\left(t\right)$, ${\hat{\theta }}_{h}\left(t\right)$ is the response value of the oscillator, and v is the proportional coefficient. The gait in this paper is not entirely consistent. Therefore, the natural frequency of the oscillator is set as a state variable, as shown below:

(19) $$\dot{\omega }\left(t\right)=v\theta_{he}\left(t\right)\cos\phi_{1}\left(t\right).$$

According to the Fourier series, the output is as follows:

(20) $$\begin{array}{l} {\hat{\theta }}_{h}\left(t\right)=\sum_{i=1}^{j}\alpha_{i}\left(t\right)\sin\phi_{i}\left(t\right)+\alpha_{0}\left(t\right)\\ {\dot{\alpha }}_{i}\left(t\right)=\eta \theta_{he}\left(t\right)\sin\phi_{i}\left(t\right) \end{array}$$

where $\alpha_{i}\left(t\right)$ is the amplitude of the output signal of the i-th oscillator, and η is the integral gain. A kernel-based nonlinear filter is introduced based on Equation (20) to achieve angle prediction, as shown below:

(21) $${\hat{\theta }}_{hf}\left(t\right)=\frac{\sum \Psi_{i}\left(\phi_{1}\left(t\right)\right)w_{i}}{\sum \Psi_{i}\left(\phi_{1}\left(t\right)\right)},$$

(22) $$\Psi_{i}\left(\phi_{1}\left(t\right)\right)=\exp\left(h\left(\cos\left(\phi_{1}\left(t\right)-c_{i}\right)-1\right)\right),$$

where $\Psi_{i}\left(\phi_{1}\left(t\right)\right)$ is the i-th kernel function, h is the width of the kernel function, $c_{i}=\frac{2\pi }{N}i$, N is the number of kernel functions, and $w_{i}$ is the weighting factor, which can be obtained through iteration as follows:

(23) $$w_{i}\left(k+1\right)=w_{i}\left(k\right)+\Psi_{i}\left(k\right)P_{i}\left(k+1\right)\left(\theta_{h}\left(k\right)-w_{i}\left(k\right)\right),$$

(24) $$P_{i}\left(k+1\right)=\frac{1}{\lambda }\left(P_{i}\left(k\right)-\frac{P_{i}^{2}\left(k\right)}{\frac{\lambda }{\Psi_{i}\left(k\right)}+P_{i}\left(k\right)}\right),$$

where P is the inverse covariance matrix and λ is a constant. The input variable in Equation (21) is converted to $\phi_{1}\left(t\right)$. $\phi_{1}\left(t\right)$ will change uniformly in the range 0−2π when Equation (21) converges. Therefore, $\phi_{1}\left(t\right)+\Delta_{\phi }$ can be used to predict human movement, which is shown as follows:

(25) $${\hat{\theta }}_{hf,\Delta_{\phi }}\left(t\right)=\frac{\sum \Psi_{i,\Delta_{\phi }}\left(\phi_{1}\left(t\right)\right)w_{i}}{\sum \Psi_{i,\Delta_{\phi }}\left(\phi_{1}\left(t\right)\right)},$$

(26) $$\Psi_{i,\Delta_{\phi }}\left(\phi_{1}\left(t\right)\right)=\exp\left(h\left(\cos\left(\phi_{1}\left(t\right)+\Delta_{\phi }-c_{i}\right)-1\right)\right).$$

Combined with the angle relationship in Figure 3, the feedforward compensation term for human motion is

(27) $${\dot{\theta }}_{hf,\Delta_{\phi }}\left(t\right)=\frac{nr_{h}}{r_{e}}\frac{d\left({\hat{\theta }}_{hf,\Delta_{\phi }}\left(t\right)-\theta_{h1,\Delta }\left(t\right)\right)}{dt}.$$

**Figure 7:**
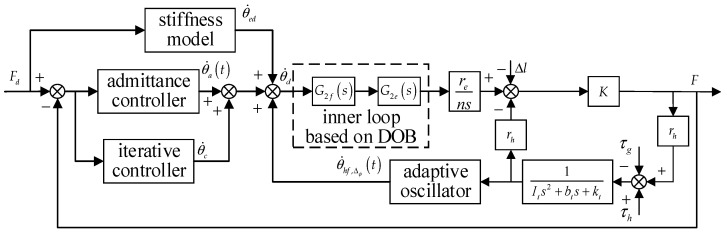
The system control block diagram.

This paper has compensated for the disturbance of the HEI and active motion to the assistance. The relationship between the inner loop and the outer loop is established by admittance control, as follows:

(28) $${\dot{\theta }}_{a}\left(t\right)=\frac{1}{I_{e}^{d}s+b_{e}^{d}}\left(F_{d}-F\right),$$

where $I_{e}^{d}$ and $b_{e}^{d}$ are virtual inertia and damping, respectively, and $F_{d}$ is the force command. Through the above analysis, the system control block diagram is shown in Figure 7.

## 4. Results

### 4.1. Test Setup

**Figure 8:**
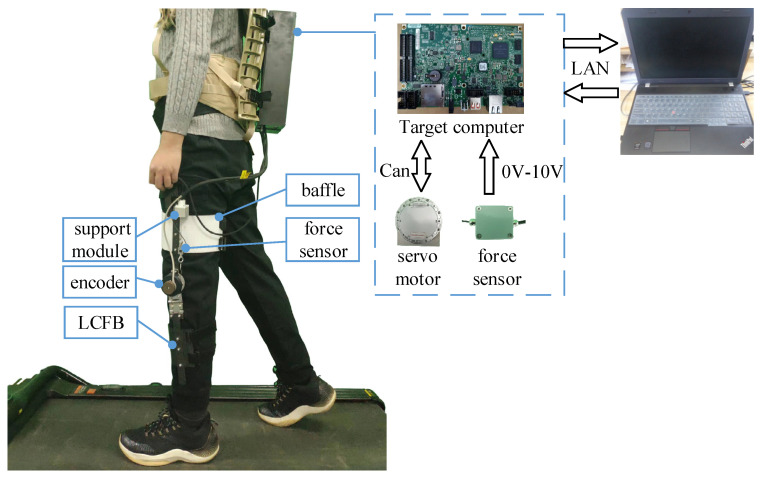
The designed knee joint exoskeleton.

The knee exoskeleton in this paper is shown in Figure 8. The designed knee joint exoskeleton is composed of the power supply, control unit, knee joint fixing structure, and sensors. The control system in this paper consists of a host computer and a target computer. The PC can be used as the host computer, through which the program is written, compiled, and downloaded to the target computer. The upper computer can also be used to control and change the variables in the target computer and receive the sensors’ values from the target computer. To improve the integration, Sbrio-9636 is adopted as the lower computer. The servo motor is a Hai Tai brushless DC motor made in Yiwu, China. The power, rated torque, rated speed, and peak torque is 200 W, 7.6 Nm, 250 RPM, and 15 Nm, respectively. The transmission ratio is 6:1. The human may be damaged when the exoskeleton severely hinders human movement during walking. Therefore, the emergency stop button is connected to the DI channel of Sbrio-9636. Subjects can press the emergency stop button in their hands when they feel inconsistent with the movement of the exoskeleton. Sbrio-9636 controls the servo motor to the initial position in time by judging that it receives the high-level signal of the emergency stop button.

The exoskeleton in this paper has the following characteristics. First, the anti-interference ability of the inner loop is poor under the disturbance of the interaction force and the friction of the Bowden cable. Second, the stiffness of the HEI changes significantly during the assisting process. Third, the dynamic performance of the outer loop is limited by the stiffness change and the frictional hysteresis of the Bowden cable. Finally, the force loading is disturbed by human movement. Combined with the above situation, the experimental ideas in this section are as follows.

According to Figure 7, the HESY contains two inputs and one output: force command, active human motion, and force response. In order to verify the influence of different input signals on the output, the human body is first fixed in a particular posture. In other words, the force loading experiment is performed while keeping the active motion signal of the human body at 0, and the constant force loading is performed during walking to verify the disturbance caused by the active movement of the human. Finally, the control method proposed in this paper is used to assist the human during walking to verify its effectiveness. Informed consent for inclusion was given by three subjects before the research, which was conducted according to the Declaration of Helsinki. The protocol of Registering Clinical Trials (ChiECRCT20200319) was approved by the Chinese Ethics Committee. The force command [33] in this section is the statistical curve of the knee joint in the flexion direction when the human is walking at a speed of 4.5 km/h.

### 4.2. PID Control in Both Inner and Outer Loop

**Figure 9:**
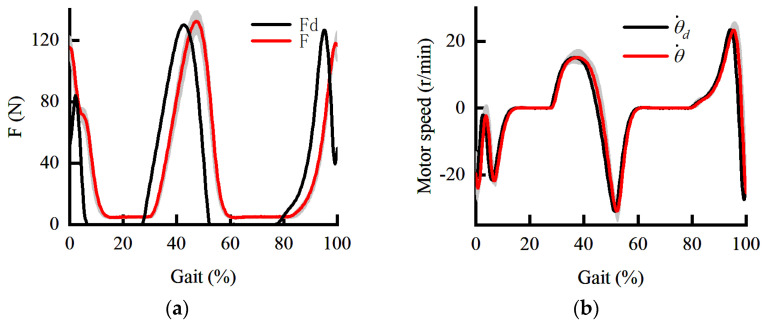
The dynamic curve under PID control. (**a**) The dynamic curve of force response; (**b**) the dynamic curve of speed response.

The posture is stable during the initial landing. Therefore, the initial landing phase is selected as the test pose. When the PID is used in both the inner and outer loops, the force load response curve and the speed response curve are shown in Figure 9. The shaded areas are the standard deviations of the force response and speed response. It can be seen from Figure 9a that there is a 60 ms delay in force response. The extension speed of the knee joint at the end of the swing phase should be reduced under the torque in the flexion direction. The protective flexion torque is rapidly increased in the initial landing phase to prevent knee hyperextension.

However, in 0–10% of the gait cycle, the assistant hardly responds when the force command changes rapidly. On the one hand, the extension of the knee joint at the end of the swing phase is hindered by inappropriate assistance. On the other hand, in order to counteract the improper assistance, the torque in the extension direction of the human is forced to increase, which is not conducive to reducing the metabolism. It can be seen from Figure 9b that the tracking performance of the inner loop is temporarily satisfactory. Therefore, the dynamic performance of force loading is limited by the outer loop. Both phase lag and amplitude attenuation in the outer loop can seriously affect the assistance to the human. Therefore, it is necessary to study the control method further to ensure the force loading performance.

### 4.3. The HEIF in the Outer Loop and the DOB in the Inner Loop

**Figure 10:**
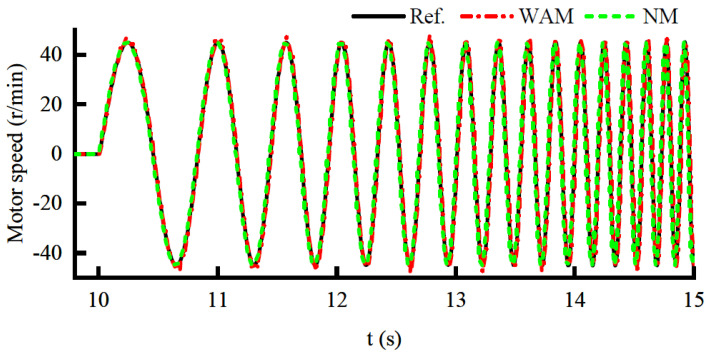
Response curve under nominal model and actual model.

The frequency sweep method is used to establish the nominal model of the actuator without load. The amplitude of the frequency sweep signal is 45 r/min, and the frequency range is 1–7 Hz. The response curve is shown in Figure 10. It can be seen that the response curve of the nominal model (NM) is basically the same as the dynamic response curve (WAM) of the actuator, which can verify the validity of the nominal model. Furthermore, the DOB can be designed based on nominal models.

Through experiments in previous studies, we have verified that the parameter perturbation of the inner loop and the poor anti-interference ability can deteriorate the dynamic performance of the outer loop [31]. Furthermore, it is concluded that the inner loop based on the DOB can improve the dynamic performance potential of the outer loop. Therefore, this paper does not repeat the DOB-based inner loop experiment. Instead, it verifies the effectiveness of the control method through combining the HEIF and the admittance control based on the DOB-based inner loop.

**Figure 11:**
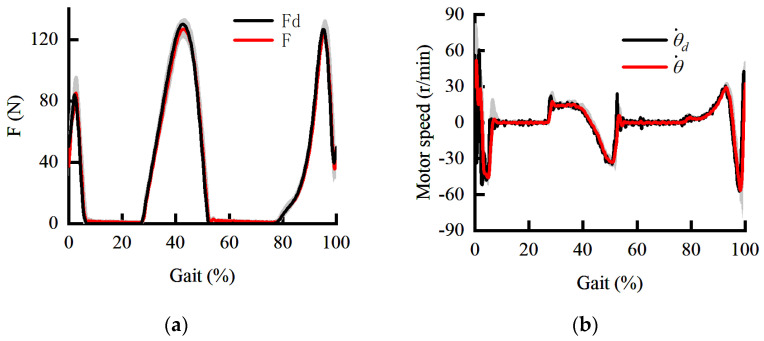
The dynamic curve under the HEIF in the outer loop and DOB in the inner loop. (**a**) The dynamic curve of force response; (**b**) the dynamic curve of speed response.

When the human is stationary, the force response and speed response curves are shown in Figure 11. The shaded areas are the standard deviations of the force response and speed response. It can be seen from Figure 11b that the inner loop still maintains adequate dynamic performance and anti-interference ability when the amplitude and the frequency of the speed command increase. The outer loop makes full use of the relationship between force and deformation to overcome the limitation of force loading caused by changes in the stiffness of the HEI. In other words, the contradiction between the stability under the maximum stiffness and the rapidity under the minimum stiffness is effectively avoided. On the other hand, the HEIF also avoids the phase lag of the force response caused by force closed-loop control and Bowden cable friction. It can be seen from Figure 11a that the phase lag and amplitude attenuation of the force response are compensated effectively when the human body is stationary. The effectiveness of the HEIF based on the DOB in the inner loop in this paper is verified. Then, we verify the interference of human motion on auxiliary accuracy and the effectiveness of the angle prediction method based on the controller of the HEIF in the outer loop and DOB in the inner loop.

**Figure 12:**
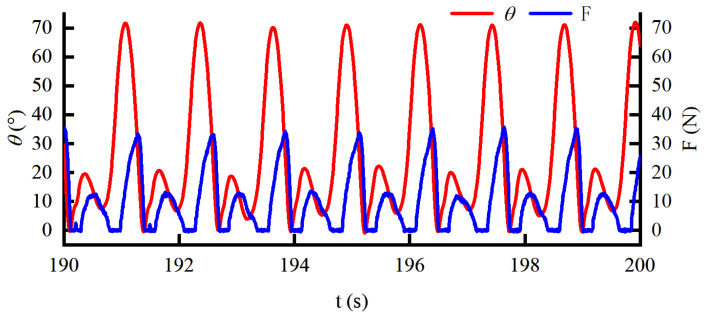
The dynamic curve for transparency detection under the HEIF in the outer loop and DOB in the inner loop during walking.

Transparency is one of the important indicators of the wearable exoskeleton. The Bowden cable can only be driven in one direction. Therefore, to verify the transparency of the exoskeleton in this paper, the force command and walking speed are set to 5 N and 4.5 km/h, respectively. The corresponding force response curve and knee displacement curve are shown in Figure 12.

It can be seen that the force response is 0 N when the knee is flexed. The transparency in the flexion direction is satisfactory. However, the assistance is disturbed by the phase lag of the actuator and the friction of the Bowden cable, when the knee joint is extended. However, the assistance is disturbed by the phase lag of the actuator and the friction of the Bowden cable when the knee joint is extended. Moreover, with the increase in the extension speed, the disturbance force also increases. The disturbance force reaches 35 N at the maximum extension speed, which severely reduces the transparency of the exoskeleton.

**Figure 13:**
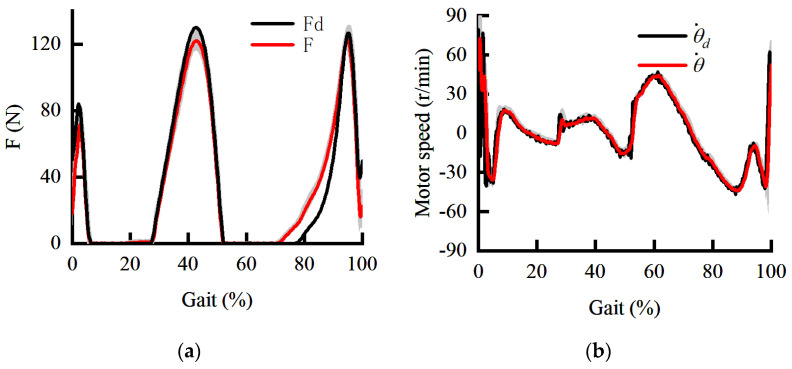
The dynamic curve under the HEIF in the outer loop and DOB in the inner loop during walking. (**a**) The dynamic curve of force response; (**b**) the dynamic curve of speed response.

Further, the force command is set as a statistical force curve, and the walking speed is 4.5 km/h. The motion information of the knee joint is compensated to the servo motor. The corresponding force response curve and the speed response curve are shown in Figure 13. The shaded areas are the standard deviations of the force response and speed response. According to Figure 13, the force response at the initial landing is only 15.9 N. Moreover, the force response is significantly disturbed in 70–90% of the gait cycle. Therefore, it is necessary to study the angle prediction method.

### 4.4. The HEIF-AO in the Outer Loop and the DOB in the Inner Loop

**Figure 14:**
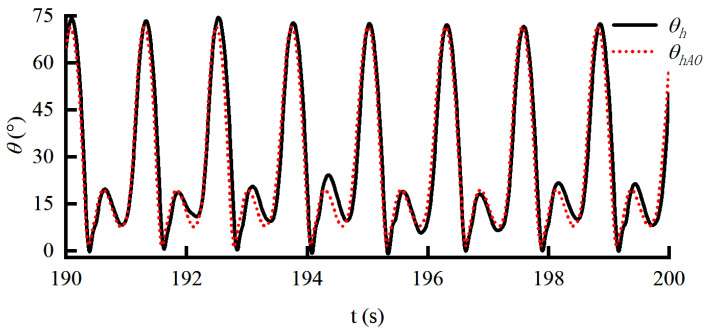
The angle curve predicted by AO.

**Figure 15:**
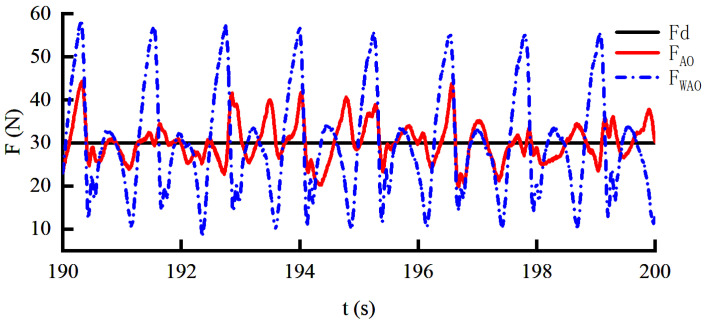
The constant force response curve under the HEIF-AO in the outer loop and the DOB in the inner loop.

A constant force of 30 N is applied to the human throughout the gait in order to intuitively verify the effectiveness of the angle prediction method while not excessively affecting human movement. The joint angle predicted by AO during walking is shown in Figure 14. It can be seen that the prediction of the joint angle can be achieved through AO. According to Figure 15, when the motion information collected by the encoder is directly compensated to the servo motor, the force response will periodically change between 10 and 60 N with the motion. According to Figure 12 and Figure 15, the range of interference force is correspondingly increased from 35 N to 50 N when the constant force is set to 5 N and 30 N, respectively. The importance of angle prediction compensation is confirmed. The force response varies between 20 and 40 N when the angle predicted by AO is input to the servo motor. The experimental results show that the AO under the constant force loading can effectively reduce the disturbance by more than 60%.

**Figure 16:**
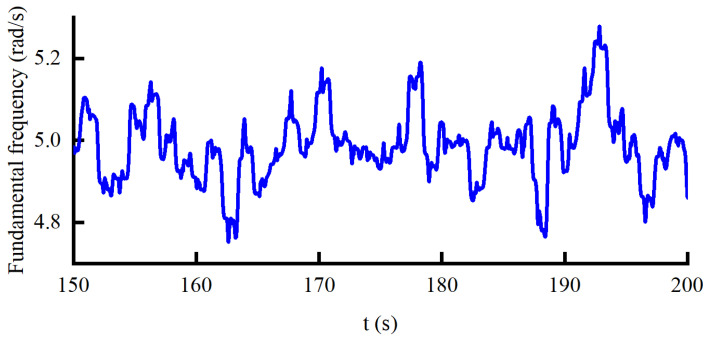
The fundamental frequency curve under the HEIF-AO in the outer loop and the DOB in the inner loop.

**Figure 17:**
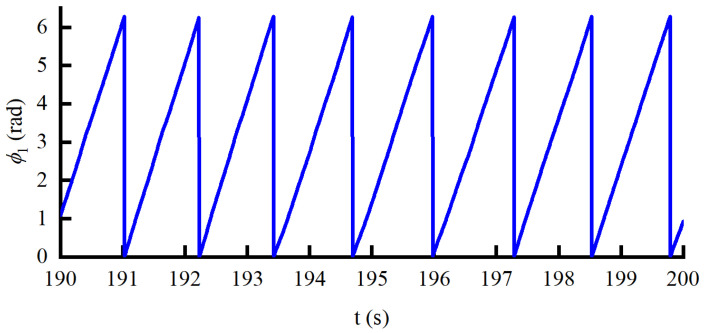
The phase curve under the HEIF-AO in the outer loop and the DOB in the inner loop.

Finally, the validity of the control method proposed in this paper is verified when the force command and the walking speed are set to a statistical force curve and 4.5 km/h, respectively. The fundamental frequency and the phase of the *i*-th oscillator curves obtained by AO are shown in Figure 16 and Figure 17, respectively. It can be seen from Figure 16 that the fundamental frequency is constantly changing between 4.7 and 5.3 rad/s. According to Figure 17, the RMSE of the phase of the *i*-th oscillator is 1.252±0.04, which shows that the motion of the knee joint is not strictly a periodic signal. Correspondingly, this is also why the local prediction bias is relatively large.

**Figure 18:**
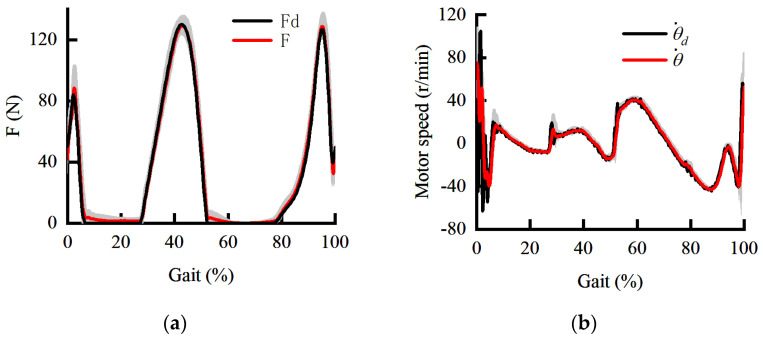
The dynamic curve under the HEIF-AO in the outer loop and the DOB in the inner loop during walking. (**a**) The dynamic curve of force response; (**b**) the dynamic curve of speed response.

The force response curve and the speed response curve are shown in Figure 18. The shaded areas are the standard deviations of the force response and speed response. It can be seen from Figure 18 that the mean value of assistance in the initial landing phase increases from 15.9 N to 32.2 N. Moreover, the assisted advancement phenomenon almost disappears in 70–90% of the gait cycle. The overall force tracking has almost no phase lag and amplitude attenuation, which can meet the assistance accuracy. The experimental results verify the validity of the theory proposed in this paper.

## 5. Conclusions

This paper analyzes the influence of Bowden cable friction, HEI, and human motion on the assistance by mathematical modeling. The corresponding control strategy is proposed based on the speed inner loop and admittance control. The experimental results show that the HEIF based on the DOB inner loop can effectively compensate for the force response’s phase lag and amplitude attenuation when the motion is ignored. However, in the initial landing phase of human walking, the average assistance is only 15.9 N. In 70%–90% of the gait cycle, the force response is significantly disturbed. The force response periodically changes between 10 and 60 N when the command is 30 N, which further verifies the disturbance of the motion to the assistance. When the predicted angle compensates for the active motion, the disturbance to the force response can be effectively reduced by more than 60%. The error of the mean assistance at the initial landing is reduced from 23.4 N to 7.1 N during walking. Moreover, in 70%–90% of the gait cycle, the disturbance to the force response is compensated. It can be proven that the theory in this paper can meet the needs of assisting knee joints.

## Abbreviations

The following abbreviations are used in this manuscript:

HESY	Human–exoskeleton system
DOB	Disturbance observer
sEMG	Surface electromyography signal
HEI	Human–exoskeleton interaction
HEIF	Human–exoskeleton interaction feedforward model
LCFB	Lightweight carbon fiber boards
AO	Adaptive oscillator
